# Measuring the reasons that discourage medical students from working in rural areas: Development and validation of a new instrument

**DOI:** 10.1097/MD.0000000000009448

**Published:** 2018-01-12

**Authors:** Sonu Goel, Federica Angeli, Neetu Singla, Dirk Ruwaard

**Affiliations:** a School of Public Health, Post Graduate Institute of Medical Education and Research, Chandigarh, India; b Department of Health Services Research, Care and Public health Research Institute (CAPHRI), Faculty of Health, Medicine and Life Sciences, Maastricht University, Maastricht; c Department of Organization Studies, Tilburg University, The Netherlands.

**Keywords:** barriers, career choice, discouraging factors, India, medical students

## Abstract

The sharply uneven distribution of human resources for health care across urban and rural areas has been a long-standing concern globally. The present study aims to develop and validate an instrument measuring the factors deterring final year students of Bachelor of Medicine and Bachelor of Surgery (MBBS) in 3 northern states of India, from working in rural areas.

The medical student's de-motivation to work in rural India (MSDRI) scale was developed using extensive literature review followed by Delphi technique. The psychometric properties of the questionnaire were assessed in terms of content validity, construct validity, data quality and reliability. Exploratory factor analysis (EFA) followed by confirmatory factor analysis (CFA) was performed to identify the primary deterrents.

Thirty-three items were generated from literature search followed by Delphi exercise. After assessing psychometric properties, the final instrument included 29 items whereas the EFA and CFA highlighted 5 main factors, namely lack of professional challenge, social segregation, socio-cultural gap, hostile professional environment, and lack of financial incentives as underpinning students’ demotivation towards working in rural areas.

The MSDRI instrument is the first valid and reliable measure for identifying deterring factors for MBBS students to work in rural areas of India. The use of it may be very helpful for policymakers as well as healthcare organizations in formulating effective measures to encourage medical students to work in rural areas, which suffer from a chronic shortage of medical personnel.

## 1 Introduction

Human resources are vital to an effective health care system.^[1]^ The World Health Organization (WHO) estimates that 57 countries worldwide are facing critical shortages of health workers.^[2]^ Adding to this, many countries are also facing rural–urban inequity in the distribution of health workers, wherein health workers are disproportionately located in urban areas. The shortage and imbalance in the distribution of human resources for health eventually lead to inequities in health services delivery and poor health outcomes of a country.^[3]^

Globally, many reasons of disparities exist between urban and rural regions. Lack of financial rewards, limited training opportunities, limited professional interaction with peers, heavy workload, social isolation, poor social services, poor living and working conditions, lack of education opportunities for children, and limited opportunities for income-generation through a second job or private practice are often cited as the main factors.^[4–7]^ If the expectations of health workers are not met then they tend to migrate within countries (to urban areas) or to developed countries.^[8–11]^ Governments in low- and high-income countries alike are struggling to attract and retain health workers in underserved areas by offering a package of incentives that covers an array of salary and non-salary related factors. Few studies have been conducted worldwide to report factors which deter medical students from working in rural areas. A study by Gadi^[4]^ in 2012 conducted in Sri Lanka and another study by Shankar and Thapa^[12]^ in Nepal reported that poor working and living conditions, fewer opportunities for postgraduate education, language differences, insecurity, lack of financial incentives were barriers identified by medical students to work in rural area.

India is also facing acute shortage of health personnel, especially in rural areas.^[13,14]^ The estimated density of health workers (allopathic physicians, nurses, and midwifes) in 2005 is 13.4, which is about half of the WHO benchmark of 25.4 workers of these categories per 10,000 population. Doctor–population ratio in rural areas in India is 3/10,000 population while it is 13/10,000 for urban areas.^[15]^ A study from the National Capital Region of India concluded that lack of infrastructural facilities, less salary, and low standard of living impedes medical students from working in rural areas.^[16]^ Another study from Bihar, central-east part of India, showed that poor living conditions, lack of professional future, priority for post-graduation, and tough working conditions were found to be the main factors for their reluctance to work in rural area.^[17]^

Most of the global literature has either considered a very limited spectrum of potential deterrents to serve in rural areas or have deployed qualitative methods of enquiry such as semi-structured questionnaires. Also, all of them have been developed for students studying in Western countries and, therefore, difficult to apply in developing countries such as India because of differences in culture and health systems.

To our knowledge, no research has comprehensively studied and validated the reasons associated with the non-selection of rural posting after Bachelor of Medicine and Bachelor of Surgery (MBBS) in India. As a consequence, validated scales for data collections are still missing as well as rigorous evidence of the factors underpinning India's sharply unequal distribution of health workforce between rural and urban areas. Analyzing the inclination of current medical students towards working in rural health care is an important exercise as they will compose the health workforce of the near future. This evidence will assist in identifying strategies to increase the quality and quantity of healthcare human resources available to underserved regions of the nation. Hence, the need for a valid and reliable scale to assess the factors deterring medical students from working in rural areas is compelling. With this background, this study aims to develop and validate a scale identifying barriers for medical students for accepting rural postings in Indian settings. If a standardized tool will be used by the researchers, the findings would become easy to compare and draw a conclusion from. This will help in mitigating the problem of human resources for health crisis.

## 2 Methods

### 2.1 Conceptual map

**Figure 1:**
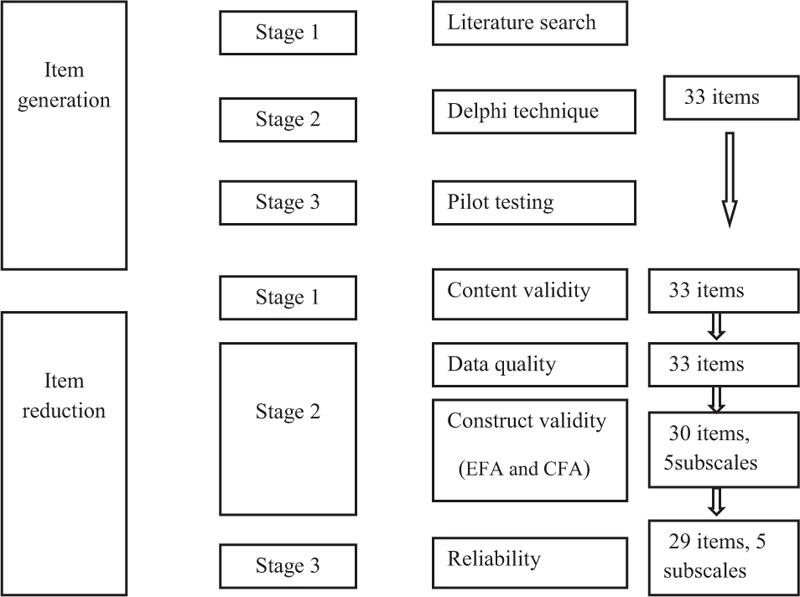
Conceptual framework for development and validation of a questionnaire measuring demotivation of medical students to work in rural areas.

A thorough literature review was carried out which was eventually narrowed down to 20 relevant studies. The final articles were used to develop a conceptual framework of the questionnaire. Delphi technique followed the literature review in which consensus on the items in the questionnaire among a group of experts was achieved. The questionnaire was pretested on 20 students to assess its correct interpretation by the respondents. Data were collected from 636 final year MBBS students and a content validity index was identified and exploratory factor analysis (EFA) and confirmatory factor analysis (CFA) were performed (Fig. 1).

### 2.2 Item generation

#### 2.2.1 Literature review

Since the diversity of each state in terms of socio-cultural differences could undermine the validity of prevalidated measurement tools existed in literature, we developed a structured questionnaire after extensive literature search using PubMed, Directory of Open Access journals, IndMED, and Google Scholar. In addition to that, a manual search of articles in Journals in the library of the Post Graduate Institute of Medical Education and Research, Chandigarh was conducted. The key words for search in various combinations included “de-motivation, medical students and interns, work in rural areas, barriers.” The search strategy had resulted in a few thousand articles, after which screening of studies was done on the basis of title and abstract followed by full article review which resulted in 20 relevant studies. The final articles were used to develop a conceptual framework of the questionnaire.

#### 2.2.2 Questionnaire development

Delphi technique followed the literature review in which consensus on the items in the questionnaire among a group of experts was achieved.^[18]^ Two rounds of Delphi were conducted. In the first round, the conceptual framework questionnaire was presented to technical experts (n = 7), public health consultants (n = 10), and public health managers (n = 5) which was followed by extensive discussions on various dimensions of the questionnaire. The first author then built consensus on the items to be included. In the second round held 2 weeks later, the experts revisited the questions, and refined wording and content of the questionnaire.

#### 2.2.3 Pilot testing of the instrument

The questionnaire was pretested on 20 students of a non-participant government medical college to assess its correct interpretation by the respondents and to identify potential problems with the methods, logistics, and the questionnaire.

### 2.3 Item reduction

#### 2.3.1 Study settings and data collection

The main study was conducted among 636 final year Bachelor of Medicine and Bachelor of Surgery (MBBS) students of 6 Government medical colleges of 3 states viz. Himachal Pradesh (HP), Punjab, and Haryana located in Northern India (2 medical colleges from each state). Final year students of MBBS were selected as they are near to complete their medical education and have to take decision about their posting in rural and urban areas. The sample size is appropriate for the study as most studies of questionnaire's validation in social sciences use 5 to 10 respondents per questionnaire item for factor analysis.^[19]^ In India, medical education consists of 5 years of medical studies followed by 1 year of clinical training (known as “internship”) in a rural or urban hospital attached to medical college. The Medical Council of India (MCI) being the statutory regulatory and registration authority for medical education and practitioners in India, has the authority to recognize a medical college or cancel its registration if the college does not comply with its guidelines.

Two trained researchers collected the data, who also had prior experience of survey research. Students were asked to rate the items that discouraged them to choose medical studies on a 5 point Likert scale where 1 represents “weakly deterrent,” 2 represents “slightly deterrent,” 3 represents “deterrent,” 4 represents “highly deterrent,” and 5 represents “strongly deterrent.” The questionnaire was handed out and collected after completion confidentially.

#### 2.3.2 Content validation and data quality

A content validity index was identified by an independent group of subject experts from state health services (different from the original panel included in the Delphi exercise) using method proposed by Lynn.^[20]^ The expert group was asked to assess the content of each item generated on a 5-point Likert scale in terms of appropriateness, comprehensibility, and clarity of phrasing of each item.

Data quality was ascertained by the completeness of responses. The percentage of missing data, extent of ceiling and floor effects, and corrected item-to-total correlation for each item in the scale were calculated. Ceiling effect in an item occurs when most of the respondents assign maximum score to that item and floor effect is in which most data points fall in the very low range of possible values. Corrected item-to-total correlations are the correlation between each item and the total score from the questionnaire and all the items should correlate with the total for a reliable scale. Items were eliminated if the missing response rate of an item was >10%, the floor and ceiling effect of an item was between 1% and 15%, and correlation of items of questionnaire <0.30 with total scale score (corrected item-to-total correlation).^[21]^

#### 2.3.3 Construct validity and reliability

Exploratory factor analysis (EFA) with varimax rotation was used to measure construct validity. It was applied on the list of selected items to group the items with similar characteristics together into factors/subscales. Multi-collinearity and singularity was checked and the items that showed high correlation (*r* = ±0.90) and low correlation (*r* = ±0.30) with other items were dropped out from further analysis.^[22]^ Kaiser–Meyer–Olkin (KMO) test was used to check sampling adequacy which should be >0.5 for a satisfactory factor analysis to proceed.^[23]^ Bartlett test was applied to check the strength of the relationship among items. The criterion of Eigenvalue ≥1 was used for defining the number of the factors that were kept.^[24–26]^ Screen plot, a graphic representation of Eigenvalues, suggested the number of the essential factors to be retained. Items were loaded into various factors on the basis of 2 criteria: each item to be included in a factor should have a factor loading >0.5 and <0.4 to the rest of the factors.^[27]^

The internal consistency of each factor was checked by calculating Cronbach *α*.^[28]^ Nunnally and Bernstein^[29]^ has indicated 0.7 to be an acceptable reliability coefficient. Convergent and discriminant validity were checked using Spearman correlation coefficient. Convergent validity is a type of construct validity which measures that the same constructs are related to each other. Conversely, discriminant validity measures that the 2 constructs are unrelated to each other. Fabrigar et al^[30]^ mention that if the correlation between an item and its factor is >0.40, it means that convergent validity exists, whereas discriminant validity exists if the correlation between an item and its factor is higher than its correlation with other factors.

Confirmatory factor analysis (CFA) was performed on EFA factors to confirm that the factor is defined according to the theoretical approach the researchers used as a starting point and to assess validity and reliability of the latent constructs. In contrast to EFA, which identifies the dimensionality of items, to drop the items having low factor loading as well as redundant items from the questionnaire; CFA is a particular case of structural equation modeling (SEM) which represents how the observed variables are interconnected.^[31]^ So, we selected the items with higher factor loadings by EFA and then performed a CFA (cross loadings are not permitted in CFA model). SEM is quite popular among the behavioral and health researchers for its ability to assess psychometric properties of measures and estimate of relationship among the constructs.^[32]^ Moreover, it can be extended to repeated measurement data, missing data, and violations of assumption of normality.^[33]^

For testing for statistical assumptions for the SEM, skewness and kurtosis values for each of the variable for assumption of normality was obtained and inspected. Multivariate outliers detection analysis was carried out using Mahalanobis distance (MD) measure with robust estimates at 97.5th quantile (*Q*) of chisquare distribution. All the samples with MD > *Q* are declared as outliers. Multicollinearity diagnostic was also carried out using variance inflation factor (VIF). Comparative fit index (CFI), Goodness-of-fit Index (GFI), Root Mean Square Error of Approximation (RMSEA), and Tucker-Lewis index (TLI) are calculated in order to measure the goodness of fit of the model. A value of about 0.06 or less for RMSEA and 0.95 or greater for CFI and TLI, 0.90 or greater for GFI, and greater than 0.80 for AGFI would indicate an acceptable model fit.^[34]^

Data were analyzed using Statistical Package for Social Sciences version—16 (SPSS IBM, New York, NY). SPSS/Amos22 has been used for CFA.

### 2.4 Ethical considerations

The study was granted ethical approval from the Institute's Ethical Committee, PGIMER, Chandigarh (PGI/IEC/2012/810-1 P-154). The approvals from Principal of selected medical college and consent of students participating in the study were obtained. To preserve the anonymity and confidentiality of participants, the respondents were asked to place the filled questionnaire in a sealed box.

## 3 Results

### 3.1 Item generation

The extensive literature review led to the development of a 33-item scale, which was named the medical student's demotivation to work in rural India (MSDRI) questionnaire to identify the reasons behind the unwillingness of medical students to work in rural areas. During the Delphi method, the wording of 2 questions was reframed, however, the number of items remained same. In pilot testing, the questionnaire was found comprehensible, correctly interpreted by students and no potential problems were found in its administration by the field investigator. No item was deleted or modified. So, the structure of the questionnaire remained the same after this stage (i.e., 33 items).

### 3.2 Item reduction

#### 3.2.1 Results on study settings and data collection

##### 3.2.1.1 Study demographics

The sample comprised 636 medical students (297 boys, 339 girls) aged 19 to 40 (mean age = 22.24 ± 1.78 years). A total of 405 (63.7%) students were born in rural areas and the majority of the students (80.8%) studied in urban areas before MBBS.

#### 3.2.2 Content validation and data quality

After extensive literature review, 33 items questionnaire was developed. Based on the expert group debate, the agreement was made that all items are contextually relevant. It was suggested that no item should be deleted from this list. Rewording of a few items was done by the experts. Data quality checks have been applied by screening of the responses of all 33 items. As the researchers were available during the process of filling out the questionnaire missing data were very negligible. No floor and ceiling effects were observed.

#### 3.2.3 Construct validity and reliability

Exploratory factor analysis was carried out with varimax rotation. KMO measure was found to be 0.930, which indicates that sample is adequate for factor analysis. Bartlett test of sphericity rejected null hypothesis at 0.05 level of significance (Bartlett test significance <0.05) and ensures the relevance of factor analysis.

Five factors (subscales) having 29 items been retained, explaining 60% of the variance, namely lack of professional challenge, social segregation, socio-cultural gap, lack of infrastructure and social support, and lack of financial incentives. Table 1 reports the results of EFA.

**Table 1:**
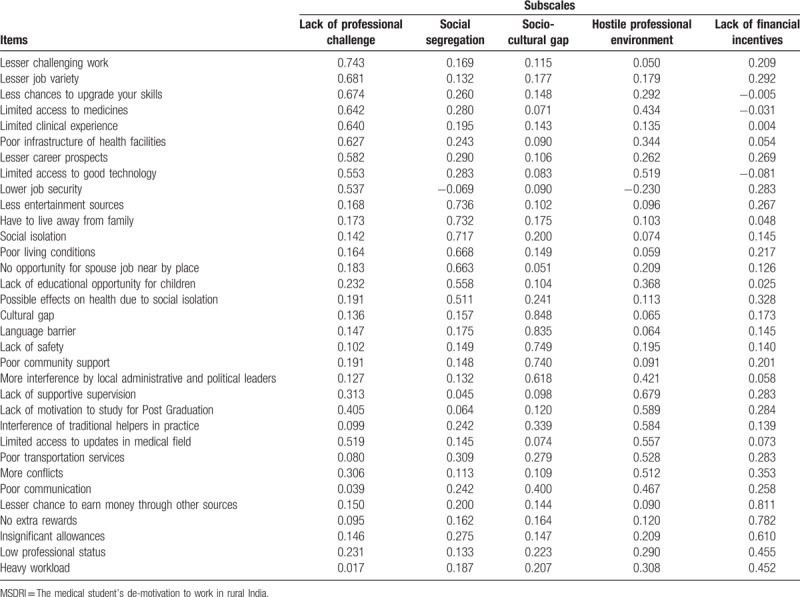
Five subscales with the corresponding loadings of items from the MSDRI questionnaire.

**Figure 2:**
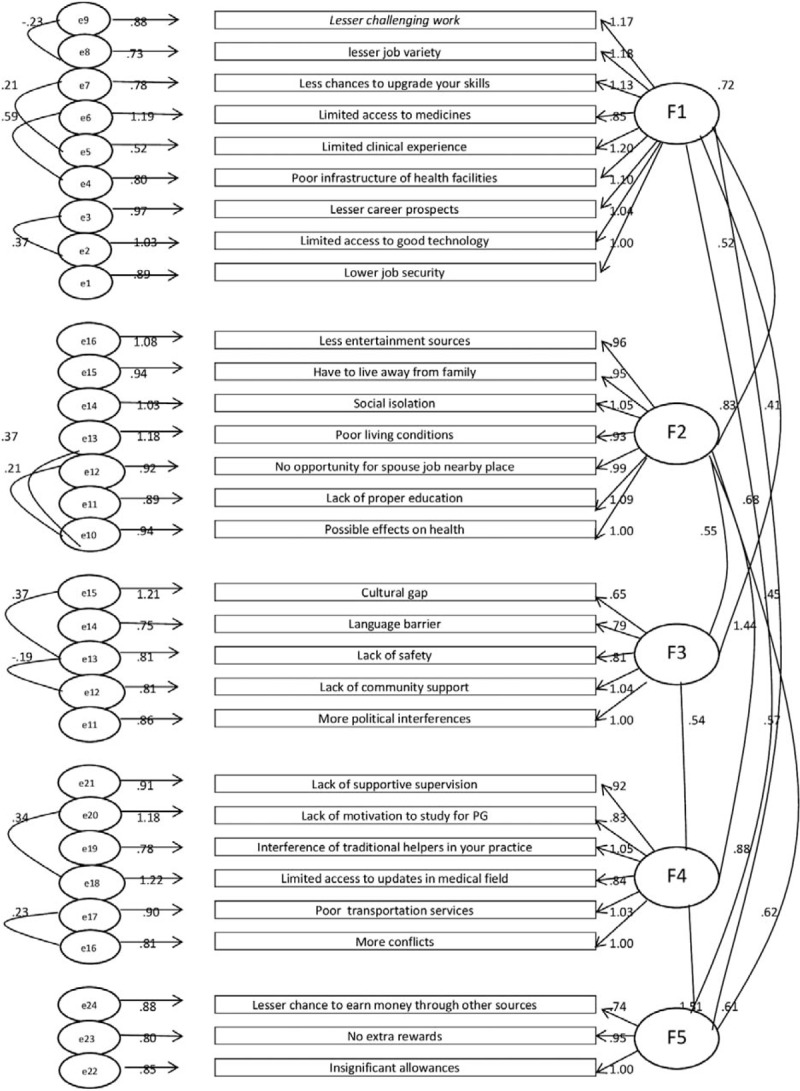
Measurement model obtained in CFA. CFA **=** confirmatory factor analysis.

Confirmatory factor analysis (CFA) was the next step performed after exploratory factor analysis to determine the factor structure of the dataset. CFA in Amos 22 produces a path diagram. Final path diagram has been shown below in Fig. 2.

The values pertaining to CFA model are goodness-of-fit index (GFI) = 0.880, adjusted goodness-of-fit index (AGFI) = 0.854, Tucker-Lewis Index (TLI) = 0.905, Comparative Fit Index (CFI) = 0.917, and RMSEA = 0.062, which indicates an acceptable model fit.

The majority of the skewness and kurtosis values for variables lie between −2 and +2, thus it is safe to assume about normality of data in a large sample size. Multivariate outliers detection analysis using Mahalanobis distance measure and Multicollinearity diagnostic using variance inflation factor (VIF) does not show any anomaly. Most of the Mahalanobis values were <3 whereas all the VIF values were <10.

The 5 factors highlighted by EFA and confirmed by CFA are the following.

### 3.3 Subscale 1: lack of professional challenge

It refers to the situation in which one's abilities and available resources in the professional environment may not be fully utilized, and where learning opportunities are limited, which hinders medical students to work in rural areas. Nine items viz. lesser challenging work, lesser job variety, lesser chances to upgrade skills, limited access to medicines, limited clinical experience, poor infrastructure of health facilities, lesser career prospects, limited access to good technology, and low job security were substantially loaded on this subscale. Internal consistency of this subscale was checked by Cronbach *α* which was 0.858 but increases to 0.897 by dropping the item low job security. Thus, this subscale consisted of 8 items.

### 3.4 Subscale 2: social segregation

The social segregation factor considers reasons which relate to the specific interrelation between the environment, family, and social life that occurs in rural settings. In particular, it points to the fact that living in rural areas is often perceived as decreasing quality of care and reducing the opportunities for nurturing private life, in terms of family bonds, friendships, and hobbies. Less entertainment sources, have to live away from family, social isolation, poor living conditions, no opportunity for spouse job at nearby place, lack of educational opportunity for children, and possible effects on health due to social isolation were 7 items substantially loaded on this subscale. Internal consistency of this subscale was checked by Cronbach *α* which was 0.859.

### 3.5 Subscale 3: socio-cultural gap

The socio-cultural gap considers the gap between the socio-cultural background of medical students and the perceived rural socio-cultural environment. Gap in cultural practices, language barrier, lack of safety, poor community support and more interference by local administrative and political leaders were loaded on the socio-cultural factors (subscale) impeding medical students to work in rural areas. Cronbach *α* measure found was 0.884.

### 3.6 Subscale 4: hostile professional environment

This subscale considers the lack of congenial professional environment in rural settings. This perception is determined by a number of different aspects, including lack of stimulating supervision, interference by local traditional health practitioners or healers, poor technological infrastructure which impairs access to medical updates and poor transportation services which compromise fast service provision and professional trips. Lack of supportive supervision, lack of motivation to study for post-graduation, interference of traditional helpers in practice, limited access to updates in medical field, poor transportation services, and more conflicts were items loaded on this subscale. Internal consistency measured by Cronbach *α* found for this subscale was 0.842.

### 3.7 Subscale 5: lack of financial incentives

The financial factor takes into account the financial considerations related to working in rural areas. The attributes lesser chances to earn money through other sources, no extra rewards and insignificant allowances have been named as the financial factors for reluctance. Internal consistency of this subscale was checked by Cronbach *α* which was 0.853. Table 1 presents the 5 subscales and the loadings on these subscales in EFA.

## 4 Discussion

The current study is the first one conducted in developing countries that developed and validated a scale describing the barriers faced by medical students for working in rural areas in Indian settings. The earlier few studies have either considered a limited spectrum of items in the questionnaire or were conducted in developed nations.^[4,12,16,17,35,36]^ After rigorous literature review and expert Delphi consultations, a questionnaire consisting of 33 items was developed which was named the MSDRI questionnaire. Interestingly, our analyses highlight 5 factors/subscales for the items listed in the questionnaire. Five main subscales, namely lack of professional challenge, social segregation, socio-cultural gap, hostile professional environment, and lack of financial incentives emerged to deter medical students from working in rural areas, and describe a complex nexus of reasons that undermines the presence of medical professionals in rural areas. Internal consistency using Cronbach *α* was found good for all subscales.

The studies conducted globally have reported the items of demotivation that fall under these new emerged subscales in the current study. Gadi,^[4]^ Kotzee and Couper,^[5]^ Manongi et al,^[6]^ Shankar and Thapa,^[12]^ Henderson and Tulloch,^[37]^ Lori et al,^[35]^ Kaye et al,^[38]^ and Steinhaeuser et al^[39]^ have mentioned items related to professional challenge as the barriers for medical students to serve in rural areas. Few studies in the context of the social segregation subscale are Gadi,^[4]^ Saini et al,^[16]^ Sinha,^[17]^ Shankar and Thapa,^[12]^ Lori et al,^[35]^ and Kaye et al^[38]^ who concluded that low standard of living impedes medical students for working in rural areas. A study by Henderson and Tulloch^[37]^ has reported that items related to hostile professional environment were responsible barriers for health workers for accepting rural postings. The health personnel value adequate water, sanitation, and up-to-date lighting and communication technologies and the lack of it hinder medical students to work in rural settings. A study by Manongi et al^[6]^ stated that forcing health workers to perform tasks beyond their scope and lack of supervision from supervisors lead to frustration and demotivation. Another study by Kaye et al^[38]^ reported that heavy workload, inadequate opportunities for continuing graduate training are the factors that make medical students not to work in rural areas. Other references in this context are Kotzee and Couper^[5]^and Saini et al.^[16]^ A study by Gadi in 2012^[4]^ stated that language differences, insecurity, and fear of an unpleasant social response, which fall under the socio-cultural gap subscale, are demotivators to medical students for working in the North East Sri Lanka.

In many studies, lack of financial incentives have been identified as a major reason for job dissatisfaction and/or migration among health workers.^[40–44]^ A study by Kaye et al^[38]^ reported insufficient salary and another study^[39]^ conducted in Germany found that low salaries and high stress levels were prime contributors to physicians migration to urban areas. Gadi,^[4]^ Saini et al,^[16]^ Shankar and Thapa^[12]^ also indicate lack of financial factors as barriers to work in rural and remote areas.

The current study had the merit of a 100% response rate and ability to capture an entire population of young medical students of 6 medical colleges of North India. Nevertheless, there are a few limitations. Our sample consists of students from medical colleges of North India which may not necessarily represent the entire medical student population of the country, because profiles of medical students and socio-environmental factors are likely to differ between different settings. Notwithstanding, the prime objective of current study was to develop and validate a contextually relevant instrument, and not underpinning various deterrents of medical students to join medical studies. However, the rural areas in most states of India are almost similar. Further, all the discouraging factors were equally weighted and some may have been disregarded, even after thorough review of literature and adopting group consensus.

In addition, there is a slightly increased risk of misguided conclusions from SEM. Acceptable fit statistics might be caused by the estimation of correlations among measurement errors and omitting relevant variables from the model. This may lead to deviation from the model applying to the true population and may also bias the parameter estimates. However, in absence of a well-defined theoretical framework which guides variable selection, we have adopted the data-driven empirical approach by correlating errors. This model may be tested by researchers in range of settings.^[45,46]^

The need for developing and validating the current instrument is especially important given an ever increasing dearth of students opting for medical studies and not choosing to work in rural areas. An increasing shortage of physicians working in rural areas and hence a decrease in population health and health inequalities will be the result. Having a newly-developed and validated instrument, like the MSDRI, to measure the reasons that discourage medical students from working in rural areas may be very helpful for policy makers. They can make new policies or strategies and set priorities so that medical students become willing to accept the rural postings. One can think of formulating effective measures such as mandatory postings in rural areas during the internship period, improved living and working conditions and providing extra incentives. Government should also make efforts in the direction of fulfilling the requirements as stated by medical students by implementing the MSDRI tool in different settings.

## 5 Conclusions

The newly-developed and validated instrument, the MSDRI, exploring the de-motivation factors of medical students for working in rural areas is a valid and reliable tool. Five broad domains emerged out in this tool are lack of professional challenge, social segregation, socio-cultural gap, hostile professional environment, and lack of financial incentives. This instrument can be implemented in other settings as well to explore the deterring factors so that strategies can be made to attract medical students to work in rural areas. However, a cautious approach should be adopted while undertaking error correlations in structural equation modeling, which may underscore the findings of study.
